# The Efficacy of Coronary Sinus Reducer in Patients with Refractory Angina: A Systematic Review and Meta-Analysis

**DOI:** 10.31083/j.rcm2503082

**Published:** 2024-03-04

**Authors:** Panagiotis Theofilis, Panayotis K Vlachakis, Marios Sagris, Emmanouil Mantzouranis, Athanasios Sakalidis, Stergios Soulaidopoulos, Christos Chasikidis, Evangelos Oikonomou, Konstantinos Tsioufis, Dimitris Tousoulis

**Affiliations:** ^1^1st Cardiology Department, “Hippokration” General Hospital, University of Athens Medical School, 11527 Athens, Greece; ^2^3rd Cardiology Department, “Sotiria” Regional Hospital for Chest Diseases, University of Athens Medical School, 11527 Athens, Greece

**Keywords:** coronary sinus reducer, refractory angina, coronary artery disease

## Abstract

**Background:**

Refractory angina is a frequently encountered phenomenon in patients with coronary artery disease, often presenting therapeutic challenges to the clinical cardiologist. Novel treatment methods have been explored in this direction, with the coronary sinus reducer (CSR) being among the most extensively-investigated.

**Methods:**

We conducted a systematic review of the literature for studies assessing the efficacy of CSR in patients with refractory angina. The primary endpoints of interest were procedural success and the improvement in angina according to the Canadian Cardiovascular Society (CCS) by at least one class. Secondary endpoints were the rate of periprocedural adverse events, the improvement by at least 2 CCS classes, and the mean change in CCS class. A random-effects meta-analysis of proportions (procedural success, improvement by ≥1 or ≥2 classes, periprocedural adverse events) or means (mean CCS class change) were performed. $I^{2}$ was chosen as the metric for between-study heterogeneity. Publication bias was assessed by the inspection of funnel plots and Egger’s regression test. We examined the risk of bias according to the Newcastle-Ottawa Scale.

**Results:**

From a total of 515 studies identified from the original search, 12 studies were finally included for data extraction. Based on their meta-analysis, we observed a high CSR procedural success (98%, 95% confidence interval (CI) 96 to 99%) with a low rate of periprocedural complications (6%, 95% CI 5 to 7%), while most patients exhibited an improvement by at least 1 CCS class (75%, 95% CI 66 to 83%) after the intervention. A significant proportion of patients demonstrated an improvement by at least 2 CCS classes (39%, 95% CI 34 to 45%), with a mean change of –1.24 CCS class (95% CI –1.40 to –1.08).

**Conclusions:**

CSR is associated with high implantation success rates and significant improvements in angina symptoms for patients with refractory angina.

## 1. Introduction

Refractory angina, a debilitating condition characterized by persistent and severe chest pain despite optimal medical therapy and revascularization interventions, remains a formidable clinical challenge [1]. It exacts a heavy toll on patients’ quality of life, limits their physical activity, and increases the burden on healthcare systems worldwide [2]. Amid this clinical conundrum, emerging interventional therapeutic approaches are being explored with varying outcomes [3]. Among them, the coronary sinus reducer has emerged as a promising intervention in difficult-to-treat situations, and its use is gaining increasing attention lately.

The coronary sinus reducer, a minimally invasive device designed to improve blood flow to the heart muscle, offers a potential ray of hope for individuals grappling with refractory angina. By redirecting venous blood from the coronary sinus into the myocardium, this innovative technology aims to alleviate angina symptoms, enhance exercise capacity, and ultimately enhance the quality of life for patients who have exhausted conventional treatment options [3].

However, before this novel intervention can be widely embraced in clinical practice, it is essential to rigorously assess its safety and efficacy. To this end, we present a comprehensive systematic review and meta-analysis, drawing from a wealth of clinical evidence, to provide a thorough evaluation of the coronary sinus reducer’s potential role in the management of refractory angina. Through a critical analysis of existing studies, we aim to offer valuable insights into the device’s clinical utility and its capacity to transform the landscape of refractory angina management.

## 2. Materials and Methods

### 2.1 Search Strategy, Inclusion and Exclusion Criteria

We conducted this systematic review and meta-analysis in accordance with the guidelines of the 2009 Preferred Reporting Items of Systematic Reviews and Meta-Analyses (PRISMA) statement (**Supplementary Table 1**) [4]. The study was pre-registered in PROSPERO (registration number: CRD42021296194).

We performed a literature search in PubMed and Scopus from inception till 9 October 2023 for articles assessing coronary sinus reducer (CSR) in patients with refractory angina. The search strategy used the following terms: (“coronary sinus reducer” OR “coronary sinus reduction” OR reducer) AND (angina OR “refractory angina” OR “coronary artery disease”). Original research articles that examined the change in anginal symptomatology following CSR were included. Studies that did not perform CSR were omitted. We further excluded all studies reporting preclinical findings, studies performed in non-ischemic cardiac disease, as well as research involving non-adult patients.

### 2.2 Data Extraction and Quality Assessment

The independent assessment of the literature search data was made by two reviewers (PT and PKV), who selected the eligible articles to be included for data extraction. In cases of discrepancies, those were resolved mutually between the two reviewers. The extracted data concerned the number of participants, the percentage of implantation success, the rate of periprocedural adverse events, the follow-up duration, the number of Canadian Cardiovascular Society (CCS) class improvement (≥1 or ≥2, mean CCS class change), as well as the mortality rate at follow-up. Additional information concerning the characteristics was retrieved, including the country of origin, mean age, sex distribution, and the inclusion criteria. The quality assessment and risk of bias assessment for the studies were conducted by the Newcastle–Ottawa Quality Assessment Scale (NOS) criteria.

### 2.3 Statistical Analysis

We performed a meta-analysis to assess the rates of procedural success and improvement in CCS class in patients receiving CSR for refractory angina. We chose $I^{2}$ and $t^{2}$ as the metrics of between-studies heterogeneity. Statistically significant heterogeneity was present in the case of $I^{2}$ values over 50%. Effect sizes were pooled via a random-effect model due to presumed variance in study design and population enrolled. The results are presented as proportions (for the procedural success, periprocedural complications, and improvement in CCS class) or means (for the mean CCS class change), with the corresponding 95% confidence intervals (CIs). Sensitivity analyses was carried out using the leave-one-out method. The possibility of publication bias was determined by funnel plot formation and inspection, as well as the performance of Egger’s regression test. All meta-analyses were generated using the meta and dmetar packages in R studio (version 2023.06.0+421, Posit Software, Boston, MA, USA).

## 3. Results

### 3.1 Study Selection

**Fig. 1.**
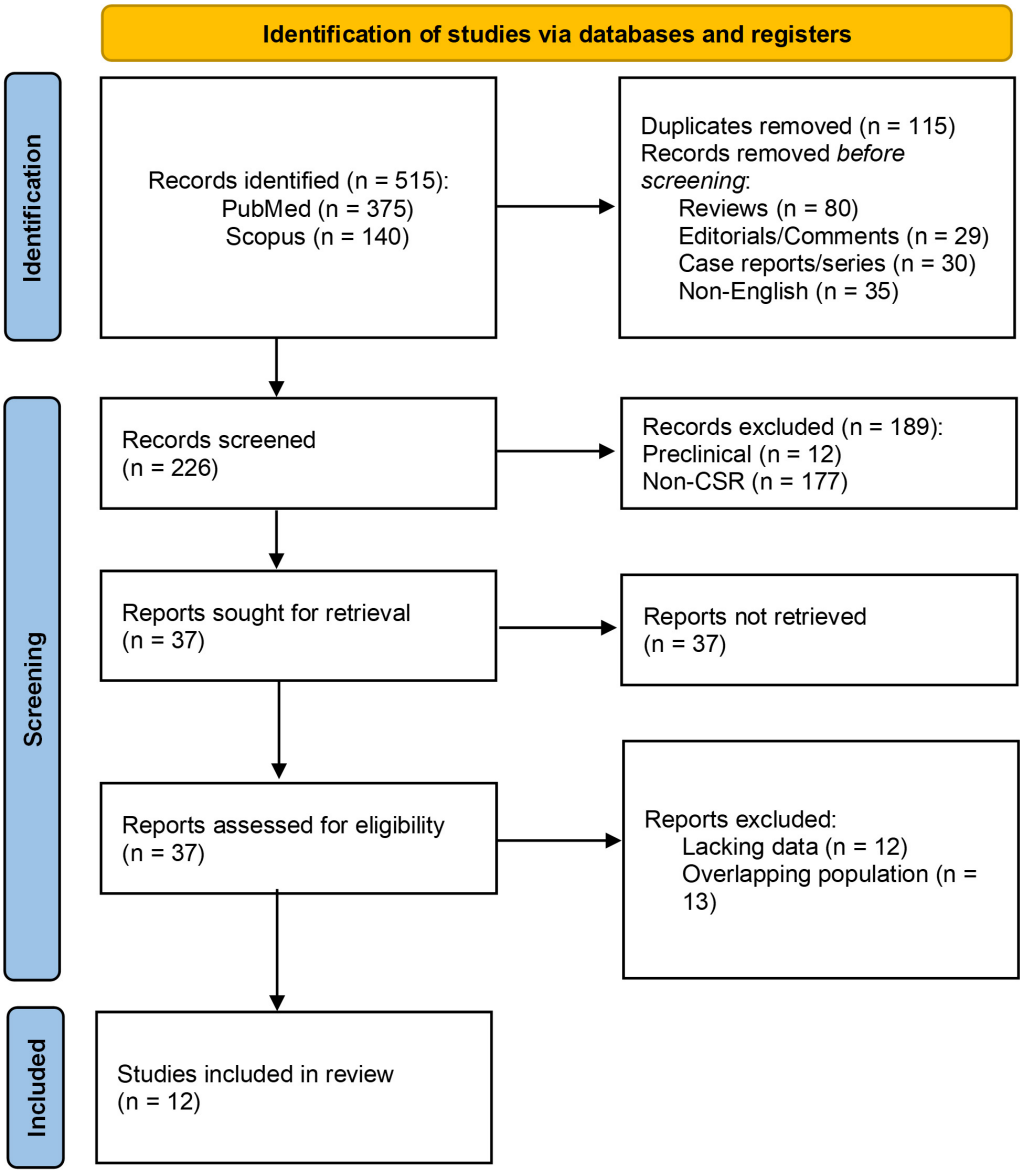
**PRISMA Flow-chart of the study selection process**. A total of 515 papers were retrieved from the initial database search. After removal of duplicates and application of exclusion criteria, 12 studies were ultimately included for data extraction and meta-analysis. CSR, coronary sinus reducer; PRISMA, Preferred Reporting Items of Systematic Reviews and Meta-Analyses.

The study selection process is illustrated in Fig. 1. The database search yielded 515 studies and, after prespecified removal of duplicates/reviews/editorials/comments/case reports and non-English articles, 226 records were screened. From those papers, 189 were further omitted after title/abstract screening due to reporting of preclinical findings or not being relevant with CSR. Thirty-seven articles were assessed for eligibility with full-text review. Ultimately, 12 studies were selected for data extraction and inclusion in the meta-analysis.

### 3.2 Study Characteristics and Quality Assessment

The characteristics of the included studies are presented in Table 1 (Ref. [5, 6, 7, 8, 9, 10, 11, 12, 13, 14, 15, 16]). Twelve studies with a total of 1679 patients undergoing CSR implantation were evaluated. Most of the included studies recruited patients with refractory angina despite optimal medical therapy who were not candidates for surgical or percutaneous revascularization, with objective evidence of myocardial ischemia. The mean age of the participants ranged from 61 to 73 years, and the predominance of male sex was evident as the percentage of men was over 70% in all studies. The rate of periprocedural adverse events was low, including device migration/dislocation/embolization, coronary sinus (CS) dissection/perforation, and access-related complications. Mortality rates varied from 0 to 17% during a follow-up period ranging from 1 to 24 months.

**Table 1.** **Baseline characteristics of the studies included in the meta-analysis**.

Study	Year	N	Country	Follow-up (months)	Mean age (years)	Males (%)	Inclusion criteria	Mortality (%)
Banai *et al*. [5]	2007	14	Germany/India	11	65	80	Refractory angina (CCS class II–IV) despite OMT, objective evidence of reversible myocardial ischemia, and LVEF >30%.	0
Konigstein *et al*. [6]	2018	48	Israel	12.5	67	83	Refractory angina (CCS class III–IV) despite OMT, objective evidence of myocardial ischemia of the left coronary arteries territory, and LVEF ≥30%.	6.3
Ponticelli *et al*. [7]	2019	50	Italy	24	61	78	Refractory angina (CCS class II–IV) despite OMT, objective evidence of myocardial ischemia of the left coronary arteries territory, CAD not amenable to PCI or CABG because of unsuitable coronary anatomy, diffuse disease, or absence of satisfactory distal graft anastomosis sites, following evaluation by the heart team.	10
D’Amico *et al*. [8]	2021	187	Italy	18.4	70	82.9	Refractory angina (CCS class II–IV) despite OMT, CAD not amenable to PCI or CABG because of unsuitable coronary anatomy, diffuse disease, or absence of satisfactory distal graft anastomosis sites, following evaluation by the heart team.	7.9
Silvis *et al*. [9]	2021	132	Netherlands	6	66	75.8	Refractory angina despite OMT, no revascularization options with PCI or CABG as decided by the local heart team, and proven stress-induced myocardial ischaemia by non-invasive stress tests.	NA
Verheye *et al*. [10]	2021	228	Multicenter	24	68	80.7	Refractory angina (CCS class II–IV) despite OMT, objective evidence of myocardial ischemia performed up to 6 months prior to consent, no revascularization options with PCI or CABG, and LVEF ≥30%.	3.5
Ponticelli *et al*. [11]	2021	658	Multicenter	16.7	70	77.8	Refractory angina (CCS class II–IV) despite OMT, objective evidence of myocardial ischemia in the left coronary artery territory, no revascularization options with PCI or CABG according to the heart team.	10.4
Vescovo *et al*. [12]	2021	219	Multicenter	13.1	69	76	Refractory angina (CCS class II–IV) despite OMT, objective evidence of inducible myocardial ischemia, no revascularization options with PCI or CABG.	17
Mrak *et al*. [13]	2022	46	Multicenter	13.2	73	91.3	Refractory angina (CCS class 2–4) despite at least 3-months OMT at maximally tolerated doses, obstructive CAD without further revascularization options, and objective evidence of reversible ischemia.	2.2
Rodríguez-Leor *et al*. [14]	2023	48	Spain	6	69	72.9	Refractory angina with no revascularization options with PCI or CABG.	2
Ferreira Reis *et al*. [15]	2022	26	Portugal	6	72	76.9	Refractory angina despite OMT, with no revascularization options.	0
Włodarczak *et al*. [16]	2023	22	Poland	1	71	86.3	Refractory angina despite OMT, with no revascularization options.	NA

CCS, Canadian Cardiovascular Society; OMT, optimal medical therapy; LVEF, left ventricular ejection fraction; PCI, percutaneous coronary intervention; CABG, coronary artery bypass grafting; CAD, coronary artery disease; NA, not available; N, number.

Overall, the quality of the studies included in the meta-analysis was found to be fair, mainly due to the lack of a control group for comparability in all of the studies, as well as the self-reported nature of the primary outcome (improvement by at least one CCS class). The detailed report of the NOS quality assessment results is presented in **Suppementary Table 2**.

### 3.3 Meta-Analysis

#### 3.3.1 Procedural Success and Periprocedural Adverse Events

**Fig. 2.**
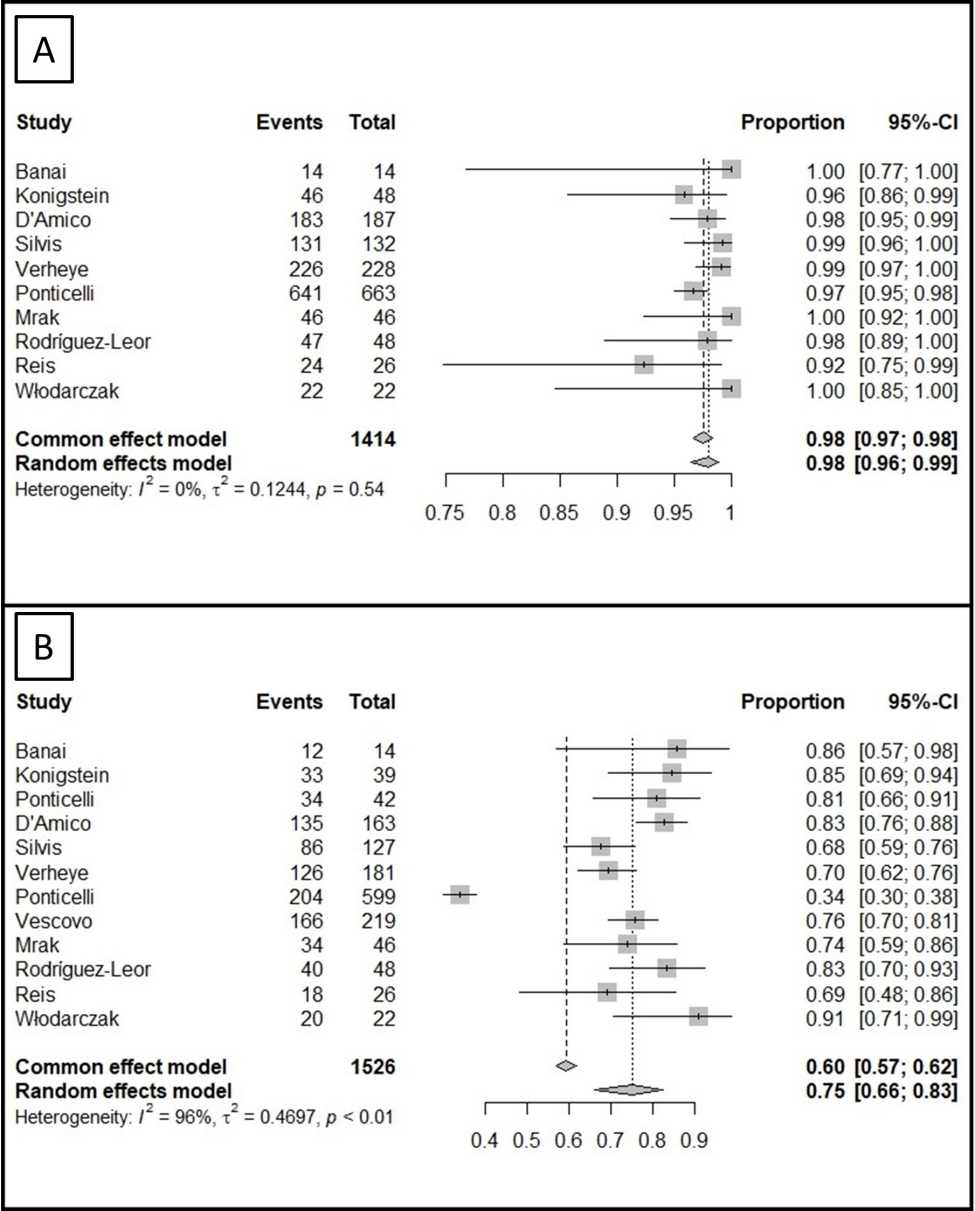
**The meta-analysis on the procedural success of CSR and the proportion of patients improving by at least one CCS class**. (A) According to the meta-analysis of proportions, a high rate of procedural success was reported in the included studies. (B) A significant proportion of patients improved by at least one CCS class at follow-up based on our meta-analysis. CSR, coronary sinus reducer; CCS, Canadian Cardiovascular Society; CI, confidence interval.

A total of 10 studies (Ref. [5, 6, 7, 8, 9, 10, 13, 14, 15, 16]) (1414 patients) reported the procedural success of CSR. According to their meta-analysis, successful implantation was reported in 98% of the cases (95% CI 96 to 99%, $I^{2}$ = 0%) (Fig. 2A). There was no evidence of asymmetry upon funnel plot inspection (**Supplementary Fig. 1**) or after performance of the Egger’s regression test (Intercept 0.60, 95% CI –0.38 to 1.58, *p* = 0.27). After exclusion of any single study, the results remained unaffected (**Supplementary Fig. 2**). The periprocedural adverse events were reported in 7 studies (1113 patients) and were noted in 6% of the cases (95% CI 5 to 7%, $I^{2}$ = 0%).

#### 3.3.2 Improvement in CCS Class

Twelve studies (Ref. [5, 6, 7, 8, 9, 10, 11, 12, 13, 14, 15, 16]) (1526 patients) assessed the improvement of at least one CCS class and found that 75% of the patients met that clinical endpoint (95% CI 66 to 83%) (Fig. 2B). High between-study heterogeneity was noted ($I^{2}$ = 96%, τ^2^ = 0.47, *p* < 0.01). Publication bias was likely according to the funnel plot (**Supplementary Fig. 3**) and the Egger’s test (Intercept 5.92, 95% CI 2.44 to 9.40, *p* = 0.008). After removal of any single study, we did not find any changes in the overall outcome (**Supplementary Fig. 4**).

**Fig. 3.**
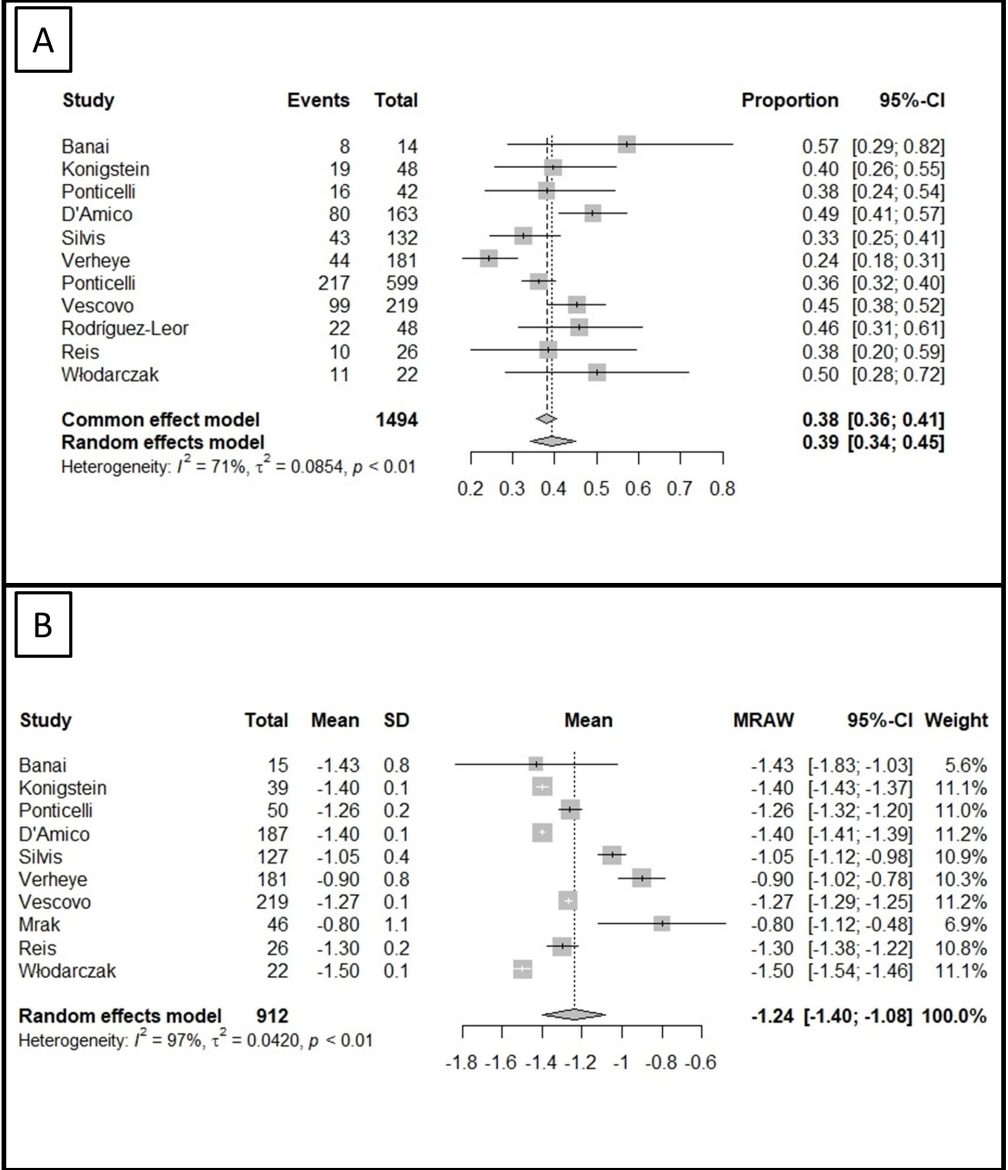
**The meta-analysis on the proportion of patients improving by at least two CCS classes and the mean change in CCS class after CSR implantation**. (A) A inegligible proportion of patients improved by at least two CCS classes at follow-up based on our meta-analysis. (B) The mean change in CCS class at follow-up was assessed by a meta-analysis of means. CSR, coronary sinus reducer; CCS, Canadian Cardiovascular Society; CI, confidence interval; MRAW, raw mean difference.

Improvement by at least two CCS classes was assessed in 11 studies (1494 patients), whose meta-analysis demonstrated that 39% of those achieved this target (95% CI 34 to 45%), with moderate between-study heterogeneity ($I^{2}$ = 71%, τ^2^ = 0.09, *p* < 0.01) (Fig. 3A). Publication bias was unlikely according to the funnel plot (**Supplementary Fig. 5**) and the Egger’s test (Intercept 0.83, 95% CI –1.41 to 3.07, *p* = 0.49). Upon sensitivity analysis, the results remained unchanged (**Supplementary Fig. 6**).

Finally, we assessed the mean change in CCS class, which was reported in 10 studies (912 patients). Based on the meta-analysis of their observations, the was a mean change of –1.24 CCS class (95% CI –1.40 to –1.08) (Fig. 3B). However, there was evidence of significant between-study heterogeneity ($I^{2}$ = 97%, τ^2^ = 0.04, *p* < 0.01), without publication bias upon funnel plot inspection (**Supplementary Fig. 7**).

## 4. Discussion

In spite of the presence of pharmacological and interventional treatments, refractory angina remains a prevalent and incapacitating clinical ailment. It stands as a substantial public health concern, adversely affecting patients’ quality of life and imposing a notable strain on healthcare resources [17]. It was in the 1950s and 1960s when Claude Beck introduced the concept of coronary sinus narrowing and performed the first surgical procedure to effectively redirect blood flow to ischemic areas of the myocardium with remarkable effectiveness [18, 19]. Since then, the CSR has emerged as a viable therapeutic option for individuals suffering from debilitating angina, especially those who have exhausted conventional medical treatments and are not suitable candidates for further revascularization procedures. In the latest chronic coronary syndromes guidelines, CSR received a IIb recommendation as a treatment option for refractory angina [20].

CSR consists of a balloon-expandable hourglass-shaped stainless steel device, with flexible longitudinal struts without welding points, and is delivered by a balloon catheter, whose front and back ends come in various sizes to accommodate the differences in the anatomy of the CS [21]. Its placement is contraindicated in patients on biventricular pacing and those with augmented right atrial pressure. Patients that are selected often are on optimal antianginal pharmacotherapy, without further targets for revascularization [21]. Moreover, the existence of ischemia in the territory of left coronary artery is frequently a prerequisite [21]. The procedure is performed under local anesthesia at the jugular vein puncture site, with the patient being on dual antiplatelet therapy together with a bolus of unfractioned heparin [21]. After placement of the CSR, a repeat venography is usually required to ascertain its position and to exclude potential complications [21]. Interestingly, according to a previous cost-effectiveness analysis, CSR appeared to be significantly associated with decreased healthcare resource use [22]. This stemed from a reduction in hospitalizations for angina, outpatient visits, the need for additional coronary angiographies, and percutaneous coronary interventions [22].

In our meta-analysis of studies investigating the utilization of the CSR in patients with refractory angina, we uncovered several key findings. Firstly, the success rate of implantation was found to be very high, exceeding 95%. Despite the “one size fits all device”, this finding indicate that the procedure is technically feasible and can be performed effectively in the majority of patients with refractory angina. Secondly, our analysis demonstrated a substantial improvement in angina symptoms among patients who underwent CSR implantation. Interestingly, approximately 75% of the patients experienced an improvement of one CCS class at the follow-up assessment. This outcome is particularly noteworthy given the subjective nature of CCS class assessment, which has been linked to adverse outcomes such as mortality and myocardial infarction in previous studies and registries [2]. Moreover, around 40% of the patients exhibited an even more remarkable improvement of two CCS classes at follow-up, moving from severe angina to mild or even no angina.

The possible mechanisms behind these positive findings are well described. Elevated backward pressure in the coronary venous system may lead to a slight dilation of arterioles, resulting in a significant reduction in vascular resistance in the subendocardium. This, in turn, enhances blood flow in the ischemic subendocardial layers of the myocardium, leading to improved contractility and a decrease in left ventricular end-diastolic pressure (LVEDP). Consequently, the decreased subendocardial vascular resistance redistributes blood from the less ischemic subepicardium to the more ischemic subendocardium, providing relief from symptoms [23, 24]. While the CCS class has its limitations, previous studies have also revealed encouraging improvements in standardized measures of angina, such as the Seattle Angina Questionnaire (SAQ) subdomains [25]. Furthermore, data from cardiopulmonary exercise testing (CPET) in these patients demonstrated an increase in anaerobic threshold during follow-up after CSR implantation, with the peak respiratory exchange ratio remaining unchanged [26]. These results suggest that the enhanced exercise capacity observed in these patients was not solely attributed to improved motivation but likely resulted from physiological changes induced by CSR implantation.

Despite these promising findings, it is crucial to acknowledge the limitations of this meta-analysis. The primary limitation lies in the nature of the included studies. The majority of the studies analyzed were single-arm studies, and only one randomized controlled trial (RCT) was available for inclusion. Furthermore, the RCT had a relatively low number of participants. The limitations associated with single-arm studies and the small sample size of the RCT may introduce bias and impact the generalizability of the results. Moreover, the placebo effect in those single-arm studies cannot be excluded. Therefore, it is important to interpret these findings with caution. Another important limitation is the lack of data on other objective measures that would be of potential interest, such as the six-minute walk test (6MWT) distance. Only 3 studies from those included reported changes in 6MWT distance, thus being impossible to conduct such a meta-analysis. Finally, a major drawback of our analysis was the significant between-study heterogeneity of the included studies. Although the study populations appeared similar in terms of inclusion criteria, mean age, and sex distribution of the participants, we assume that additional important confounding factors may have contributed. However, we should also state that the results remained unaffected after the sensitivity analysis.

Looking ahead, we anticipate the results of the COSIRA II (Efficacy of the COronary SInus Reducer in Patients with Refractory Angina II) trial (NCT05102019), which is currently in the recruitment phase. This larger RCT has the potential to provide more robust evidence regarding the efficacy and safety of CSR implantation for refractory angina and possibly upgrade its recommendation in this patient population. Moreover, larger-scale observational studies are also underway (NCT01566175, NCT02710435) to improve our understanding on the efficacy and safety of this intervention. Finally, the use of this treatment could be expanded to patients with microvascular angina in case the COronary SInus Reducer for the Treatment of Refractory Microvascular Angina (COSIMA) trial ends up with positive results (NCT04606459).

## 5. Conclusions

In conclusion, the meta-analysis of available studies indicates that coronary sinus reducer implantation is associated with high success rates and significant improvements in angina symptoms for patients with refractory angina. However, the limitations of the current evidence and unanswered questions, such as the use of this device regardless of their specific antianginal therapy, highlight the need for further research. If the positive trends observed in this meta-analysis are confirmed, coronary sinus reducer therapy may hold significant clinical implications for the management of refractory angina, offering new hope for patients with this challenging condition.

## Data Availability

The datasets supporting this article are available upon reasonable request from the corresponding author.

## Supplementary Material

Supplementary material associated with this article can be found, in the online version, at https://doi.org/10.31083/j.rcm2503082.
